# Impact of the frequency of plasma viral load monitoring on treatment outcomes among children with perinatally acquired HIV

**DOI:** 10.1002/jia2.25312

**Published:** 2019-06-09

**Authors:** Tavitiya Sudjaritruk, David C Boettiger, Lam Van Nguyen, Thahira J Mohamed, Dewi K Wati, Torsak Bunupuradah, Rawiwan Hansudewechakul, Penh S Ly, Pagakrong Lumbiganon, Revathy A Nallusamy, Moy S Fong, Kulkanya Chokephaibulkit, Nik K Nik Yusoff, Khanh H Truong, Viet C Do, Annette H Sohn, Virat Sirisanthana, the TREAT Asia Pediatric HIV Observational Database of IeDEA Asia‐Pacific, J Tucker, N Kumarasamy, E Chandrasekaran, D Vedaswari, IB Ramajaya, N Kurniati, D Muktiarti, M Lim, F Daut, P Mohamad, MR Drawis, KC Chan, L Aurpibul, R Hansudewechakul, P Ounchanum, S Denjanta, A Kongphonoi, P Kosalaraksa, P Tharnprisan, T Udomphanit, G Jourdain, T Puthanakit, S Anugulruengkit, W Jantarabenjakul, R Nadsasarn, K Lapphra, W Phongsamart, S Sricharoenchai, QT Du, CH Nguyen, TM Ha, VT An, DTK Khu, AN Pham, LT Nguyen, ON Le, Ho Chi, JL Ross, T Suwanlerk, MG Law, A Kariminia

**Affiliations:** ^1^ Department of Pediatrics Faculty of Medicine Chiang Mai University Chiang Mai Thailand; ^2^ Research Institute for Health Sciences Chiang Mai University Chiang Mai Thailand; ^3^ The Kirby Institute UNSW Australia Sydney Australia; ^4^ National Hospital of Pediatrics Hanoi Vietnam; ^5^ Pediatric Institute, Hospital Kuala Lumpur Kuala Lumpur Malaysia; ^6^ Sanglah Hospital Udayana University Bali Indonesia; ^7^ HIV‐NAT The Thai Red Cross AIDS Research Centre Bangkok Thailand; ^8^ Chiangrai Prachanukroh Hospital Chiang Rai Thailand; ^9^ National Centre for HIV/AIDS, Dermatology and STDs Phnom Penh Cambodia; ^10^ Department of Pediatrics Faculty of Medicine Khon Kaen University Khon Kaen Thailand; ^11^ Penang Hospital Penang Malaysia; ^12^ Hospital Likas Kota Kinabalu Malaysia; ^13^ Department of Pediatrics Faculty of Medicine Siriraj Hospital Mahidol University Bangkok Thailand; ^14^ Hospital Raja Perempuan Zainab II Kelantan Malaysia; ^15^ Children's Hospital 1 Ho Chi Minh City Vietnam; ^16^ Children's Hospital 2 Ho Chi Minh City Vietnam; ^17^ TREAT Asia/amfAR – The Foundation for AIDS Research Bangkok Thailand

**Keywords:** antiretroviral treatment, viral load, monitoring, paediatric, treatment failure, Asia

## Abstract

**Introduction:**

Recommendations on the optimal frequency of plasma viral load (pVL) monitoring in children living with HIV (CLWH) who are stable on combination antiretroviral therapy (cART) are inconsistent. This study aimed to determine the impact of annual versus semi‐annual pVL monitoring on treatment outcomes in Asian CLWH.

**Methods:**

Data on children with perinatally acquired HIV aged <18 years on first‐line, non‐nucleoside reverse transcriptase inhibitor‐based cART with viral suppression (two consecutive pVL <400 copies/mL over a six‐month period) were included from a regional cohort study; those exposed to prior mono‐ or dual antiretroviral treatment were excluded. Frequency of pVL monitoring was determined at the site‐level based on the median rate of pVL measurement: annual 0.75 to 1.5, and semi‐annual >1.5 tests/patient/year. Treatment failure was defined as virologic failure (two consecutive pVL >1000 copies/mL), change of antiretroviral drug class, or death. Baseline was the date of the second consecutive pVL <400 copies/mL. Competing risk regression models were used to identify predictors of treatment failure.

**Results:**

During January 2008 to March 2015, there were 1220 eligible children from 10 sites that performed at least annual pVL monitoring, 1042 (85%) and 178 (15%) were from sites performing annual (n = 6) and semi‐annual pVL monitoring (n = 4) respectively. Pre‐cART, 675 children (55%) had World Health Organization clinical stage 3 or 4, the median nadir CD4 percentage was 9%, and the median pVL was 5.2 log_10_ copies/mL. At baseline, the median age was 9.2 years, 64% were on nevirapine‐based regimens, the median cART duration was 1.6 years, and the median CD4 percentage was 26%. Over the follow‐up period, 258 (25%) CLWH with annual and 40 (23%) with semi‐annual pVL monitoring developed treatment failure, corresponding to incidence rates of 5.4 (95% CI: 4.8 to 6.1) and 4.3 (95% CI: 3.1 to 5.8) per 100 patient‐years of follow‐up respectively (*p *=* *0.27). In multivariable analyses, the frequency of pVL monitoring was not associated with treatment failure (adjusted hazard ratio: 1.12; 95% CI: 0.80 to 1.59).

**Conclusions:**

Annual compared to semi‐annual pVL monitoring was not associated with an increased risk of treatment failure in our cohort of virally suppressed children with perinatally acquired HIV on first‐line NNRTI‐based cART.

**Abbreviations**
aHR: adjusted subdistribution hazard ratio
BMI: body mass index
cART: combination antiretroviral therapy
CLWH: children living with HIV
DHHS: The US Department of Health and Human Services
HAZ: height‐for‐age z‐score
HR: subdistribution hazard ratio
IeDEA: International Epidemiology Databases to Evaluate AIDS
IQR: interquartile range
NNRTI: non‐nucleoside reverse transcriptase inhibitor
NRTI: nucleoside reverse transcriptase inhibitor
PENTA: the Paediatric European Network for Treatment of AIDS
pVL: plasma viral load
PYFU: patient‐years of follow‐up
TApHOD: the TREAT Asia Pediatric HIV Observational Database
WAZ: weight‐for‐age z‐score
WHO: World Health Organization

## 1 Introduction

In 2017, there were an estimated 1.8 million children younger than 15 years of age living with HIV (CLWH) worldwide, of whom 110,000 were in the Asia‐Pacific region 1. Globally, 52% of CLWH were on combination antiretroviral therapy (cART) compared to 59% of adults 2.

Routine laboratory monitoring, including plasma viral load (pVL) and CD4 cell count, can confirm successful responses to cART 3, 4, 5. However, implementation of these strategies may be impeded by costs and availability of resources, particularly in resource‐limited settings. For those who are clinically, immunologically and virologically stable on cART, the frequency of laboratory monitoring may be reduced. However, recommendations for pVL monitoring in cART‐treated CLWH with viral suppression are inconsistent 3, 4, 5. The US Department of Health and Human Services (DHHS) treatment guidelines recommend performing routine pVL testing every three to four months among children who are stable on long‐term cART 3. Similarly, the Paediatric European Network for Treatment of AIDS (PENTA) guidelines recommend obtaining pVL testing every three to four months once children have been established on treatment 4. The World Health Organization (WHO) recommendations suggest annual pVL monitoring when children are clinically stable and virally suppressed on cART 5.

Because of the resources needed and financial costs associated with pVL testing, it is important to confirm whether less frequent monitoring strategies are safe for subsets of cART‐treated children. This study aimed to determine the impact of annual versus semi‐annual pVL monitoring on treatment outcomes within a cohort of Asian children with perinatally acquired HIV infection who were stable on first‐line, non‐nucleoside reverse transcriptase inhibitor (NNRTI)‐based cART.

## 2 Methods

### 2.1 Study cohort and population

The TREAT Asia Pediatric HIV Observational Database (TApHOD) is a multinational, longitudinal observational cohort of CLWH in the Asia‐Pacific region that is part of the International Epidemiology Databases to Evaluate AIDS (IeDEA) Asia‐Pacific. The data collection methods have been previously described 6. In March 2015, TApHOD included data obtained from 5783 CLWH who had ever received treatment at one of the 16 university‐based or public paediatric HIV programmes in Cambodia (n = 1), India (n = 1), Indonesia (n = 2), Malaysia (n = 4), Thailand (n = 5) or Vietnam (n = 3). TREAT Asia/amfAR (Bangkok, Thailand) is the coordinating centre for the cohort, and the Kirby Institute, University of New South Wales (Sydney, Australia) is the data management and statistical analysis centre. Ethics approval for this study was obtained at all participating sites, the study coordinating centre, and the data management and statistical analysis centre. Participant consent is deferred to the individual participating sites and their institutional review boards.

The study population consisted of CLWH enrolled in the cohort before March 2015. All children with perinatally acquired HIV aged <18 years on a virally suppressive first‐line NNRTI‐based cART regimen were included in the study. This was defined as having two or more consecutive pVL tests <400 copies/mL spanning a period of at least six months while on a first cART regimen containing one NNRTI and at least two nucleoside reverse transcriptase inhibitors (NRTIs). We included only CLWH on NNRTI‐based regimens as this is the predominant first‐line regimen combination in the region. CLWH exposed to a mono‐ or dual‐antiretroviral regimen for the purposes of treatment prior to cART initiation and those receiving care at sites without at least annual pVL monitoring were excluded (Figure S1).

### 2.2 Frequency of pVL monitoring

The frequency of pVL monitoring, which was largely influenced by the national HIV programme policy of each site, was determined at the site‐level based on a median rate of routine pVL measurement over the cohort follow‐up period (Table 1). Sites were designated as having annual monitoring if their median rate of pVL measurement ranged between 0.75 and 1.5 tests/patient/year, whereas sites were categorized as having semi‐annual monitoring if their median rate was >1.5 tests/patient/year. Participants from the sites with a median pVL measurement rate <0.75 tests/patient/year over the entire follow‐up period in the cohort were defined as having less than annual monitoring and were excluded.

**Table 1:** Paediatric site‐specific frequencies of pVL monitoring

Site name	Frequency of pVL monitoring (test/patient/year)		Number of children included	Monitoring frequency category^a^
	Median	IQR		
Thailand #1	2.8	2.3 to 3.4	109	Semi‐annual
Malaysia #1	2.8	2.3 to 3.2	45	Semi‐annual
Malaysia #2	2.5	2.2 to 2.6	9	Semi‐annual
Malaysia #3	2.2	1.7 to 2.4	15	Semi‐annual
Thailand #2	1.4	1.1 to 1.8	86	Annual
Thailand #3	1.2	0.9 to 1.7	128	Annual
Thailand #4	1.2	0.9 to 1.6	188	Annual
Thailand #5	1.1	0.8 to 1.4	371	Annual
Cambodia #1	1.1	0.9 to 1.3	229	Annual
Malaysia #4	1.0	0.7 to 1.2	40	Annual
Vietnam #1	0.7	0.5 to 1.0	‐	Less than annual (excluded)
Vietnam #2	0.5	0.3 to 0.8	‐	Less than annual (excluded)
Indonesia #1	0.5	0.3 to 0.8	‐	Less than annual (excluded)
Vietnam #3	0.5	0.2 to 0.8	‐	Less than annual (excluded)
Indonesia #2	0.3	0.2 to 0.4	‐	Less than annual (excluded)
India	0.2	0.1 to 0.3	‐	Less than annual (excluded)

IQR, interquartile range; pVL, plasma viral load.

a Less than annual, <0.75 tests/patient/year; Annual, 0.75 to 1.5 tests/patient/year; Semi‐annual, >1.5 tests/patient/year.

### 2.3 Outcome definitions

In this study, treatment failure was defined as either having two consecutive pVL >1000 copies/mL while on cART, a change of antiretroviral drug class (e.g. change from NNRTI‐based to protease inhibitor‐based regimens) which was considered as a surrogate parameter of treatment failure in our CLWH with established viral suppression over a six‐month period, or death (e.g. all‐cause mortality). Loss to follow‐up was described as not presenting to the clinic for longer than 12 months without documentation of transfer to another clinic or death.

### 2.4 Data collection

Clinical characteristics were extracted from the database. Baseline was considered the date of the second pVL measurement <400 copies/mL to meet criteria for viral suppression. The window periods for measurements of weight, height, CD4 and pVL were within plus or minus three months of the date of interest. The measurement taken closest to that date was used in the analysis. Weight‐for‐age z‐scores (WAZ) were calculated using the 1977 National Center for Health Statistics/WHO reference 7. Height‐for‐age (HAZ) and body mass index (BMI)‐for‐age z‐scores were calculated using the 2007 WHO growth references 8. Nadir CD4 percentage and cell count were defined as the lowest CD4 values documented before treatment initiation. CLWH were considered having hepatitis B or C virus coinfection if they had an evidence of a positive hepatitis B surface antigen, or a positive hepatitis C antibody test respectively. Orphan status, school attendance, WHO clinical stage, cART regimens and HIV disclosure status were based on the last available recorded status as of the date of interest.

### 2.5 Statistical analysis

Since this was a retrospective observational cohort study, we did not conduct sample size and power calculations, according to the Strengthening the Reporting of Observational Studies in Epidemiology guidelines. Clinical characteristics of CLWH were analysed using descriptive statistics. The incidence rates of treatment failure were calculated by dividing the number of participants with treatment failure by the total number of patient‐years of follow‐up (PYFU). Kaplan‐Meier estimates and the log‐rank test were used to describe and compare the cumulative probability of treatment failure by the frequency of pVL monitoring.

Competing risk regression models were used to analyse independent factors associated with treatment failure. CLWH were censored at their last clinic visit if they did not demonstrate evidence of treatment failure over the follow‐up. Loss to follow‐up was considered a competing risk event. Covariates were included in the multivariable model if any category exhibited a *p *<* *0.10 on univariable analysis, together with the predictors of interest identified *a priori*. Covariates were retained in the final model if one or more categories exhibited a *p *<* *0.05. Associations of predictor and study outcome were summarized with subdistribution hazard ratios (HR) and adjusted subdistribution hazard ratios (aHR) for univariable and multivariable analyses respectively. CLWH with missing covariate data were included in all analysis, but HR and aHR for the missing categories were not reported. Stata statistical software, version 14.1 (StataCorp LP, College, Station, TX) was used to perform all statistical analysis.

## 3 Results

### 3.1 Cohort characteristics

Among 2413 children with perinatally acquired HIV infection who had started cART at one of the 10 sites performing at least annual pVL monitoring, 1220 (46.7%) children from Cambodia, Malaysia, and Thailand met eligibility criteria (Figure S1). The median follow‐up time (interquartile range; IQR) of the analysis cohort was 4.6 (2.2 to 6.6) years. Of all eligible children, 1042 (85.4%) were from six sites which performed annual pVL monitoring while 178 (14.6%) were from four sites which performed semi‐annual pVL monitoring (Table 1). The overall median frequency of pVL monitoring (IQR) was 1.5 (1.1 to 2.1) tests/patient/year.

Of the 1220 eligible children, 581 (47.6%) were male. Prior to cART initiation, 55.3% of children were classed as WHO clinical stage 3 or 4, 77.9% had a nadir CD4 percentage <15%, and 62.5% had pVL >5.0 log_10_ copies/mL. At baseline, the median age (IQR) was 9.2 (6.3 to 12.0) years, and 786 (64.4%) were on nevirapine‐based regimens. The median cART duration and CD4 percentage (IQR) were 1.6 (1.0 to 3.0) years and 26% (20% to 31%) respectively (Table 2). Over the study period, there were 20 children (1.6%) lost to follow‐up; all were among individuals with annual pVL monitoring.

**Table 2:** Characteristics of Asian children with perinatally acquired HIV infcetion on first‐line, NNRTI‐based regimens, stratified by the frequency of pVL monitoring

Characteristics^a^^,^ b	Total number of children	Frequency of pVL monitoring	
		Annual (n = 1042)	Semi‐annual (n = 178)
Demographic characteristics			
Age, years	1220	9.6 (6.6 to 12.2)	7.5 (4.5 to 10.0)
Male sex	1220	490 (47.0)	91 (51.1)
Orphan status	1051		
Both parents alive		211 (22.1)	37 (38.1)
Single parent alive		274 (28.7)	24 (24.7)
Neither parent alive		469 (49.2)	36 (37.1)
Attending school	1022	747 (80.8)	53 (54.6)
Anthropometric parameters			
HAZ	1175	−2.0 (−2.8 to −1.2)	−1.5 (−2.2 to −0.8)
>−1.5		339 (33.6)	85 (51.2)
−1.5 to −2.5		337 (33.4)	53 (31.9)
<−2.5		333 (33.0)	28 (16.9)
WAZ	1179	−1.8 (−2.7 to −1.0)	−1.4 (−2.4 to −0.6)
>−1.5		409 (40.4)	99 (59.6)
−1.5 to −2.5		302 (29.8)	31 (18.7)
<−2.5		302 (29.8)	36 (21.7)
BMI‐for‐age z‐score	1174	−0.6 (−1.3 to 0.1)	−0.5 (−1.2 to 0.3)
>0		284 (28.2)	57 (34.5)
0 to −1.5		534 (52.9)	80 (48.5)
<−1.5		191 (18.9)	28 (17.0)
Concurrent medical illness			
Had hepatitis B coinfection	813	41 (6.0)	4 (3.2)
Had hepatitis C coinfection	459	9 (2.2)	0 (0.0)
HIV‐specific characteristics prior to cART initiation			
The most severe WHO clinical stage	1220		
Stage 1 and 2		461 (44.2)	84 (47.2)
Stage 3		327 (31.4)	44 (24.7)
Stage 4		254 (24.4)	50 (28.1)
Nadir CD4 percentage, %	1213	8 (2 to 14)	12 (4 to 20)
>15		202 (23.6)	66 (37.1)
5 to 15		470 (55.0)	64 (36.0)
<5		363 (42.4)	48 (26.9)
Nadir CD4 cell count^c^, cell/mm^3^	1027	156 (37 to 358)	216 (48 to 478)
>350		231 (25.6)	42 (33.4)
200 to 350		161 (17.9)	24 (19.0)
<200		509 (56.5)	60 (47.6)
pVL, log_10_ copies/mL	529	5.3 (4.9 to 5.7)	5.0 (4.8 to 5.6)
<4.3		37 (9.4)	21 (15.2)
4.3 to 5.0		94 (24.1)	46 (33.3)
>5.0		260 (66.5)	71 (51.5)
HIV‐specific characteristics			
Frequency of pVL measurement, times/patient/year	1220	1.4 (1.1 to 1.8)	2.5 (2.2 to 3.1)
HIV status disclosed	759	274 (41.4)	20 (20.6)
Current NNRTI‐based cART regimen	1220		
Nevirapine‐based		689 (66.1)	97 (54.5)
Efavirenz‐based		353 (33.9)	81 (45.5)
Duration of NNRTI‐based cART use, years	1220	1.8 (1.0 to 3.0)	1.0 (0.9 to 1.8)
Current CD4 percentage, %	1190	26 (20 to 31)	26 (21 to 32)
>30		258 (25.4)	49 (28.2)
20 to 30		527 (51.9)	90 (51.7)
<20		231 (22.7)	35 (20.1)
Current CD4 cell count^c^, cells/mm^3^	1001	693 (492 to 982)	630 (455 to 893)
>750		384 (43.7)	51 (42.2)
500 to 750		267 (30.3)	32 (26.4)
<500		229 (26.0)	38 (31.4)

BMI, body mass index; cART, combination antiretroviral therapy; HAZ, height‐for‐age z‐score; HIV, human immunodeficiency virus; IQR, interquartile range; NNRTI, non‐nucleoside reverse transcriptase inhibitor; pVL, plasma viral load; WAZ, weight‐for‐age z‐score; WHO, World Health Organization.

^a^Characteristics were evaluated at baseline (the date of the second plasma viral load measurement <400 copies/mL), unless otherwise specified; ^b^data are presented as n (%) for categorical data and median (IQR) for continuous data; ^c^data on CD4 cell count were based on children and adolescents aged greater than five years.

### 3.2 Incidence of treatment failure

Over the follow‐up, 298 (24.4%) children met criteria for treatment failure, including 77 (5.9%) with two consecutive pVL >1000 copies/mL while on cART, 211 (17.3%) with cART drug class change, and 10 (0.8%) deaths. The overall incidence rate for treatment failure was 5.2 (95% CI: 4.7 to 5.9) events per 100 PYFU.

**Figure 1:**
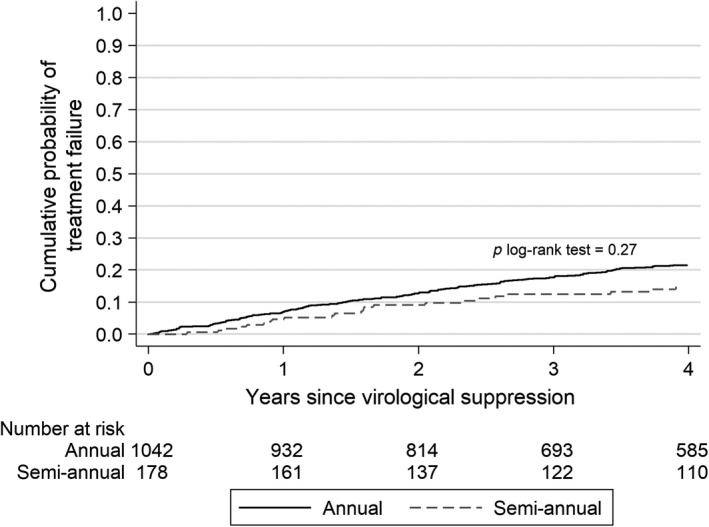
The Kaplan‐Meier estimates of unadjusted cumulative probability for treatment failure by plasma viral load monitoring frequency during the first four years of follow‐up.

Treatment failure developed in 258 (24.8%) children at sites with annual pVL monitoring, corresponding to an incidence rate of 5.4 (95% CI: 4.8 to 6.1) events per 100 PYFU; failure was detected among 40 (22.5%) children at sites with semi‐annual monitoring, corresponding to an incidence rate of 4.3 (95% CI: 3.1 to 5.8) events per 100 PYFU (*p* log‐rank test = 0.27). The Kaplan‐Meier estimates of unadjusted cumulative probability for treatment failure by pVL monitoring frequency during the first four years of follow‐up are illustrated in Figure 1.

### 3.3 Characteristics of children with treatment failure

Of the 298 children with treatment failure, 133 (44.6%) were male. At the time of treatment failure, the median age (IQR) was 12.6 (10.3 to 15.1) years and CD4 percentage was 26% (19 to 31%). Among 89 children who were performed pVL measurement when developing treatment failure, the pVL (IQR) was 4.2 (3.4 to 4.8) log_10_ copies/mL. The characteristics of children without treatment failure and children who were lost to follow‐up are summarized in Table 3.

**Table 3:** Characteristics of Asian children with perinatally acquired HIV infection with and without treatment failure and children with loss to follow‐up

Characteristics^a^	Children with treatment failure (n = 298)	Children without treatment failure at censoring (n = 902)	Children with loss to follow‐up (n = 20)
Demographic characteristics			
Age, years	12.6 (10.3 to 15.1)	14.8 (11.4 to 17.4)	15.0 (10.8 to 16.3)
Male sex	133 (44.6)	437 (48.4)	11 (55.0)
Anthropometric parameters			
HAZ	1.8 (−2.4 to −1.1)	−1.5 (−2.2 to −0.8)	−1.8 (−2.2 to −0.7)
>−1.5	104 (34.9)	300 (33.3)	4 (20.0)
−1.5 to −2.5	109 (36.6)	211 (23.4)	5 (25.0)
<−2.5	60 (20.1)	106 (11.8)	2 (10.0)
Unknown	25 (8.4)	285 (31.6)	9 (45.0)
WAZ	−1.7 (−2.5 to −0.9)	−1.6 (−2.4 to −0.7)	−2.0 (−3.4 to −1.2)
>−1.5	121 (40.6)	305 (33.8)	4 (20.0)
−1.5 to −2.5	82 (27.5)	186 (20.6)	3 (15.0)
<−2.5	61 (20.5)	153 (17.0)	5 (25.0)
Unknown	34 (11.4)	258 (28.6)	8 (40.0)
BMI‐for‐age z‐score	−0.8 (−1.3 to −0.1)	−0.8 (−1.6 to −0.1)	−1.2 (−1.5 to −0.7)
>0	56 (18.8)	129 (14.3)	0 (0)
0 to −1.5	153 (51.3)	311 (34.5)	8 (40.0)
<−1.5	52 (17.4)	158 (17.5)	3 (15.0)
Unknown	37 (12.4)	304 (33.7)	9 (45.0)
HIV‐specific characteristics			
Current CD4 percentage, %	26 (19 to 31)	30 (25 to 34)	26 (25 to 28)
>30	77 (25.8)	220 (24.4)	1 (5.0)
20 to 30	120 (40.3)	218 (24.2)	7 (35.0)
<20	74 (24.8)	42 (4.7)	12 (60.0)
Unknown	27 (9.1)	422 (46.8)	0 (0)
Current CD4 count^b^, cell/mm^3^	641 (433 to 938)	719 (539 to 975)	638 (575 to 947)
>750	103 (35.8)	227 (25.5)	3 (15.0)
500 to 750	75 (26.0)	177 (19.9)	5 (25.0)
<500	84 (29.2)	93 (10.5)	1 (5.0)
Unknown	26 (9.0)	392 (44.1)	11 (55.0)
Current pVL, log_10_ copies/mL	4.2 (3.4 to 4.8)	Suppressed	Suppressed

BMI, body mass index; HAZ, height‐for‐age z‐score; IQR, interquartile range; pVL, plasma viral load; WAZ, weight‐for‐age z‐score.

^a^Data are presented as n (%) for categorical data and median (IQR) for continuous data; ^b^data on CD4 cell count were based on children and adolescents aged greater than 5 years.

### 3.4 Predictors of treatment failure

In the multivariable analysis, compared with semi‐annual pVL monitoring, annual monitoring was not associated with treatment failure. However, older age and WHO clinical stage 4 prior to cART initiation (compared with WHO clinical stage 1 and 2) significantly increased the risk of treatment failure. Additionally, longer duration of NNRTI‐based cART use was associated with a decreased risk of treatment failure (Table 4).

**Table 4:** Predictors of treatment failure among Asian children with perinatally acquired HIV infection

Characteristics^a^	Treatment failure, n (%)	Patient years of follow‐up	Rate per 100 patient‐years (95% CI)	Univariable analysis		Multivariable analysis	
				Crude HR (95% CI)	*p* value	Adjusted HR (95% CI)	*p* value
Frequency of pVL monitoring							
Semi‐annual	40 (22.5%)	937.0	4.27 (3.13 to 5.82)	Ref		Ref	
Annual	258 (24.8%)	4751.8	5.43 (4.81 to 6.13)	1.19 (0.86 to 1.65)	0.29	1.12 (0.80 to 1.59)	0.50
Age (per 1 year increased)	298 (24.4%)	5688.8	5.24 (4.68 to 5.87)	1.09 (1.06 to 1.12)	<0.01	1.11 (1.07 to 1.14)	<0.01
Sex							
Male	133 (22.9%)	2686.5	4.95 (4.18 to 5.87)	Ref		‐	‐
Female	165 (25.8%)	3002.2	5.50 (4.72 to 6.40)	1.11 (0.89 to 1.40)	0.36		
Orphan status					<0.01^b^		0.23^b^
Both parents alive	36 (14.5%)	1121.2	3.21 (2.32 to 4.45)	Ref		Ref	
Single parent alive	64 (21.5%)	1320.0	4.85 (3.79 to 6.19)	1.52 (1.01 to 2.28)	0.05	1.18 (0.78 to 1.79)	0.43
Neither parent alive	138 (27.3%)	2272.5	6.07 (5.14 to 7.18)	1.92 (1.34 to 2.76)	<0.01	1.38 (0.94 to 2.02)	0.10
Unknown	60 (35.5%)	975.1	6.15 (4.78 to 7.93)	‐		‐	
School attendance							
Attended	203 (25.4%)	3631.4	5.59 (4.87 to 6.41)	Ref		Ref	
Not attended	30 (13.5%)	973.4	3.08 (2.15 to 4.41)	0.53 (0.36 to 0.77)	<0.01	0.78 (0.52 to 1.17)	0.23
Unknown	65 (32.8%)	1084.0	6.00 (4.70 to 7.65)	‐		‐	
HIV disclosure status							
Disclosed	112 (24.1%)	2042.1	5.48 (4.56 to 6.60)	Ref		Ref	
Not disclosed	97 (33.0%)	1017.5	9.53 (7.81 to 11.63)	1.65 (1.25 to 2.18)	<0.01	1.24 (0.88 to 1.75)	0.21
Unknown	89 (19.3%)	2629.2	3.39 (2.75 to 4.17)	‐		‐	
HAZ					0.03^b^		0.36^b^
>−1.5	86 (20.3%)	1915.1	4.49 (3.64 to 5.55)	Ref		Ref	
−1.5 to −2.5	92 (23.6%)	1885.7	4.88 (3.98 to 5.98)	1.09 (0.81 to 1.46)	0.56	0.94 (0.70 to 1.27)	0.71
<−2.5	107 (29.6%)	1751.2	6.11 (5.06 to 7.38)	1.37 (1.03 to 1.82)	0.03	1.14 (0.85 to 1.52)	0.40
Unknown	13 (28.9%)	136.7	9.51 (5.52 to 16.38)	‐		‐	
WAZ					0.36^b^		
>−1.5	110 (21.7%)	2357.4	4.67 (3.87 to 5.62)	Ref		‐	‐
−1.5 to −2.5	90 (27.0%)	1587.4	5.67 (4.61 to 6.97)	1.20 (0.91 to 1.59)	0.19		
<−2.5	86 (25.4%)	1627.7	5.28 (4.28 to 6.53)	1.13 (0.85 to 1.49)	0.41		
Unknown	12 (29.3%)	116.2	10.33 (5.86 to 18.18)	‐			
BMI‐for‐age z‐score					0.42^b^		
>0	80 (23.5%)	1608.4	4.97 (4.00 to 6.19)	Ref		‐	‐
0 to −1.5	146 (23.8%)	2943.0	4.96 (4.22 to 5.83)	1.00 (0.76 to 1.31)	1.00		
<−1.5	59 (26.9%)	999.3	5.90 (4.57 to 7.62)	1.16 (0.84 to 1.62)	0.37		
Unknown	13 (28.3%)	138.0	9.42 (5.47 to 16.22)	‐			
Hepatitis B coinfection				
No hepatitis B coinfection	195 (25.4%)	3406.6	5.72 (4.97 to 6.59)	Ref		Ref	
Had hepatitis B coinfection	18 (40.0%)	196.7	9.15 (5.76 to 14.52)	1.58 (0.99 to 2.55)	0.06	1.39 (0.88 to 2.20)	0.16
Unknown	85 (20.9%)	2085.4	4.08 (3.30 to 5.04)	‐			
Hepatitis C coinfection				
No hepatitis C coinfection	145 (32.2%)	1922.4	7.54 (6.41 to 8.88)	Ref		‐	‐
Had hepatitis C coinfection	3 (33.3%)	52.1	5.76 (1.86 to 17.85)	0.79 (0.27 to 2.29)	0.66		
Unknown	150 (19.7%)	3714.2	4.04 (3.44 to 4.74)	‐			
Nadir CD4 percentage prior to cART initiation, %					0.35^b^		
>15	59 (22.0%)	1124.3	5.25 (4.07 to 6.77)	Ref		‐	‐
5 to 15	124 (23.2%)	2515.9	4.93 (4.13 to 5.88)	0.96 (0.70 to 1.32)	0.81		
<5	114 (27.7%)	1991.2	5.73 (4.77 to 6.88)	1.13 (0.82 to 1.55)	0.45		
Unknown	1 (14.3%)	57.4	1.74 (0.25 to 12.38)	‐			
pVL prior to cART initiation, log_10_ copies/mL					0.08^b^		0.13^b^
<4.3	18 (31.0%)	306.4	5.88 (3.70 to 9.32)	Ref		Ref	
4.3 to 5.0	37 (26.4%)	745.7	4.96 (3.59 to 6.85)	0.83 (0.48 to 1.44)	0.52	0.77 (0.44 to 1.33)	0.35
>5.0	72 (21.8%)	1876.3	3.84 (3.05 to 4.83)	0.66 (0.40 to 1.09)	0.11	0.69 (0.41 to 1.15)	0.16
Unknown	171 (24.7%)	2760.4	6.19 (5.33 to 7.20)	‐		‐	
WHO clinical stage prior to cART initiation					0.01^b^		<0.01^b^
Stage 1 and 2	115 (21.1%)	2610.9	4.40 (3.67 to 5.29)	Ref		Ref	
Stage 3	95 (25.6%)	1655.8	5.74 (4.69 to 7.02)	1.26 (0.96 to 1.65)	0.09	1.31 (0.99 to 1.73)	0.06
Stage 4	88 (28.9%)	1422.1	6.19 (5.02 to 7.63)	1.41 (1.07 to 1.87)	0.02	1.43 (1.08 to 1.88)	0.01
Current NNRTI‐based cART regimen							
Nevirapine‐based	178 (22.6%)	3598.0	4.95 (4.27 to 5.73)	Ref		‐	‐
Efavirenz‐based	120 (27.6%)	2090.7	5.74 (4.80 to 6.86)	1.17 (0.93 to 1.48)	0.17		
Duration of NNRTI‐based cART use (per 1 year increased)	298 (24.4%)	5688.8	5.24 (4.68 to 5.87)	0.92 (0.85 to 1.00)	0.05	0.85 (0.77 to 0.93)	<0.01
Current CD4 percentage, %					<0.01^b^		0.59^b^
>30	59 (19.2%)	1371.8	4.30 (3.33 to 5.55)	Ref		Ref	
20 to 30	145 (23.5%)	2842.4	5.10 (4.34 to 6.00)	1.21 (0.90 to 1.64)	0.21	1.00 (0.73 to 1.36)	0.99
<20	86 (32.3%)	1348.1	6.38 (5.16 to 7.88)	1.63 (1.16 to 2.27)	<0.01	1.09 (0.75 to 1.58)	0.65
Unknown	8 (26.7%)	126.5	6.32 (3.16 to 12.64)	‐		‐	

BMI, body mass index; cART, combination antiretroviral therapy; HAZ, height‐for‐age z‐score; HIV, human immunodeficiency virus; NNRTI, non‐nucleoside reverse transcriptase inhibitors; pVL, plasma viral load; WAZ, weight‐for‐age z‐score; WHO, World Health Organization.

^a^Characteristics were evaluated at baseline (the date of the second plasma viral load measurement <400 copies/mL), unless otherwise specified; ^b^overall *P* for trend for ordinal variables and overall *P* for heterogeneity for nominal variables.

## 4 Discussion

We observed no substantial differences in treatment failure rates on the basis of annual versus semi‐annual pVL monitoring in a regional cohort of Asian CLWH with viral suppression on first‐line, NNRTI‐based regimens. Older age, severe WHO clinical stage prior to cART initiation, and shorter duration of NNRTI‐based cART use were the strongest predictors of treatment failure in this population.

HIV treatment guidelines vary in how frequently pVL testing is recommended. The DHHS treatment guidelines and the International Diseases Society of America recommend that pVL testing should be performed at four to eight weeks intervals after cART initiation, but can be extended to semi‐annually for adherent, clinically stable adolescents and adults whose viral load has been suppressed (pVL < 20 copies/mL) for at least two years and whose immunologic status has been consistently stable on cART (CD4 count >300 cells/mm^3^) 9, 10. The International Antiviral Society‐USA Panel Recommendations suggest that pVL monitoring in adults can be prolonged from every three months to semi‐annual testing once the viral suppression has been maintained (pVL < 50 copies/mL) for at least a year in the context of consistent adherence to cART 11. However, the guidelines for paediatric HIV management of the US DHHS and PENTA still recommend pVL testing, every three to four months, regardless of whether children have been virologically and immunologically stable on long‐term cART, in order to provide enhanced adherence monitoring or disease progression 3, 4. In contrast, WHO guidelines allow for annual pVL measurement once children are well established on cART 5.

pVL testing is a gold standard for HIV treatment monitoring. The elevation of pVL among patients who achieved viral suppression after cART initiation is an indicator of adherence problems, which may need support interventions, or true treatment failure because of HIV drug resistance mutations, which may require cART regimen changes 12, 13. However, in resource‐limited settings, pVL testing can be expensive, with the cost for reagents alone ranging from 10 to 85 US dollars/test 14. Thus, reducing the frequency of pVL testing from semi‐annual to annual for sub‐populations where this is appropriate could contribute to substantial country‐level cost savings over time in low‐ and middle‐income settings in Asia. This would potentially allow for these resources to be reallocated to other services that improve the quality of comprehensive care for CLWH, or for adherence support for those who are not stable on treatment.

We found that older age significantly increased the risk of treatment failure in our cohort. Since adolescence is a vulnerable period, several challenges they experience may result in adherence problems and eventually treatment failure, especially for those with perinatally acquired HIV infection who started cART at very young ages 15, 16. Additionally, advanced HIV disease before cART initiation was associated with treatment failure, which has been observed in other studies 17, 18. Longer duration on first‐line NNRTI regimens appeared to be a protective factor against treatment failure. While substantially longer cART into adolescence may be associated with higher risk of failure, the majority of our cohort included CLWH who were still on their first‐line regimens and in paediatric care.

Although our study was strengthened by its regional scope and duration of follow‐up, it did not involve a randomized comparison or study‐specified monitoring, requiring the use of a site‐level definition for the pVL monitoring frequency based on the median testing rate at the site. This risks misclassification bias among sites which were defined as having annual monitoring but whose upper IQR of pVL testing frequency overlap with the threshold for semi‐annual monitoring (Thailand #2, #3 and #4). There is also the potential for misclassification at the individual level if patients had more frequent testing due to clinical concerns relative to their site classification. The relatively small sample size, particularly in the semi‐annual group, might also bias our results towards the null effect. We excluded CLWH receiving care at sites with less than 0.75 tests/patient/year, which biases our results towards facilities with greater resources to perform pVL measurement. Specifically, our Indian, Indonesian and Vietnamese cohort sites were excluded, further limiting the generalizability of our data within the region. Furthermore, we restricted the treatment outcomes we assessed to virologic failure, change of cART drug class and death, and did not evaluate opportunistic infections, hospitalizations and HIV drug resistance mutations, which may be impacted by poor adherence and viral failure at levels below our definitions. Although we relied on pVL testing as our primary means of assessing treatment response and failure, we did not have self‐reported adherence data to compare pVL to as part of our analysis.

## 5 Conclusions

Among this cohort of Asian children with perinatally acquired HIV who were stable on suppressive first‐line NNRTI‐based cART, annual pVL monitoring was not associated with an increased risk of treatment failure compared with semi‐annual monitoring. Taking into consideration patient‐level factors when determining pVL monitoring frequency may help balance clinical needs and program costs.

## Competing interests

AHS has received travel and project funding to her institution from ViiV Healthcare. Other authors declare no conflict of interest related to this study.

## Authors’ contributions

TS developed the conception of research, designed the study, and developed the protocol. TS, LVN, TJM, DKW, TB, RH, PSL, PL, RAN, MSF, KC, NNY, KHT and VCD performed the data collection. DCB and TS designed and conducted the statistical analyses. TS contributed to the interpretation of study results. TS wrote the first draft of manuscript. DCB, LVN, TJM, DKW, AHS and VS provided critical revisions to the manuscript. TS revised the manuscript. All authors reviewed and approved the final version of the manuscript.

## Supporting information


**Figure S1.** The schema of study participants.*Viral suppression after cART initiation was defined as having two or more consecutive pVL tests <400 copies/mL spanning a period of at least six months. cART, combination antiretroviral therapy; NNRTI, non‐nucleoside reverse transcriptase inhibitor; pVL, plasma viral load; TApHOD, TREAT Asia Pediatric HIV Observational Database.

## Appendix 1 The TREAT Asia Pediatric HIV Network

### 1.1

PS Ly (TApHOD Steering Committee member), and V Khol, National Centre for HIV/AIDS, Dermatology and STDs, Phnom Penh, Cambodia; J Tucker, New Hope for Cambodian Children, Phnom Penh, Cambodia; N Kumarasamy (TApHOD Steering Committee member), and E Chandrasekaran, YRG CARE Medical Centre, CART CRS, Chennai, India; DK Wati (TApHOD Steering Committee member), D Vedaswari, and IB Ramajaya, Sanglah Hospital, Udayana University, Bali, Indonesia; N Kurniati (TApHOD Steering Committee member), and D Muktiarti, Cipto Mangunkusumo – Faculty of Medicine Universitas Indonesia, Jakarta, Indonesia; SM Fong (TApHOD Steering Committee member), M Lim, and F Daut, Hospital Likas, Kota Kinabalu, Malaysia; NK Nik Yusoff (TApHOD Steering Committee member; Current Steering Committee Chair), and P Mohamad, Hospital Raja Perempuan Zainab II, Kelantan, Malaysia; TJ Mohamed (TApHOD Steering Committee member), and MR Drawis, Pediatric Institute, Hospital Kuala Lumpur, Kuala Lumpur, Malaysia; R Nallusamy (TApHOD Steering Committee member), and KC Chan, Penang Hospital, Penang, Malaysia; T Sudjaritruk (TApHOD Steering Committee member), V Sirisanthana, and L Aurpibul, Department of Pediatrics, Faculty of Medicine, and Research Institute for Health Sciences, Chiang Mai University, Chiang Mai, Thailand; R Hansudewechakul (TApHOD Steering Committee member), P Ounchanum, S Denjanta, and A Kongphonoi, Chiangrai Prachanukroh Hospital, Chiang Rai, Thailand; P Lumbiganon (TApHOD Steering Committee member), P Kosalaraksa, P Tharnprisan, and T Udomphanit, Division of Infectious Diseases, Department of Pediatrics, Faculty of Medicine, Khon Kaen University, Khon Kaen, Thailand; G Jourdain, PHPT‐IRD UMI 174 (Institut de recherche pour le développement and Chiang Mai University), Chiang Mai, Thailand; T Puthanakit (TApHOD Steering Committee member), S Anugulruengkit, W Jantarabenjakul and R Nadsasarn, Department of Pediatrics, Faculty of Medicine and Research Unit in Pediatric and Infectious Diseases, Chulalongkorn University, Bangkok, Thailand; K Chokephaibulkit (TApHOD Steering Committee member; co‐Chair), K Lapphra, W Phongsamart, and S Sricharoenchai, Department of Pediatrics, Faculty of Medicine Siriraj Hospital, Mahidol University, Bangkok, Thailand; KH Truong (TApHOD Steering Committee member), QT Du, and CH Nguyen, Children's Hospital 1, Ho Chi Minh City, Vietnam; VC Do (TApHOD Steering Committee member), TM Ha, and VT An Children's Hospital 2, Ho Chi Minh City, Vietnam; LV Nguyen (TApHOD Steering Committee member), DTK Khu, AN Pham, and LT Nguyen, National Hospital of Pediatrics, Hanoi, Vietnam; ON Le, Worldwide Orphans Foundation, Ho Chi Minh City, Vietnam; AH Sohn (TApHOD Steering Committee member), JL Ross, and T Suwanlerk, TREAT Asia/amfAR – The Foundation for AIDS Research, Bangkok, Thailand; MG Law (TApHOD Steering Committee member) and A Kariminia, The Kirby Institute, UNSW Australia, Sydney, Australia.
