# Supplementary material for: Impact of the frequency of plasma viral load monitoring on treatment outcomes among children with perinatally acquired HIV
Source: J Int AIDS Soc. 2019 Jun 9;22(6):e25312. doi: 10.1002/jia2.25312 (PMC6556679; doi:10.1002/jia2.25312)
Supplement: Supplementary file 1 — Figure S1. The schema of study participants.*Viral suppression after cART initiation was defined as having two or more consecutive pVL tests <400 copies/mL spanning a period of at least six months. cART, combination antiretroviral therapy; NNRTI, non‐nucleoside reverse transcriptase inhibitor; pVL, plasma viral load; TApHOD, TREAT Asia Pediatric HIV Observational Database. [file JIA2-22-e25312-s001.docx]

**Supplemental figure. The schema of study participants.**

Abbreviations: cART, combination antiretroviral therapy; NNRTI, non-nucleoside reverse transcriptase inhibitor; pVL, plasma viral load; TApHOD, TREAT Asia Pediatric HIV Observational Database.

^*^Viral suppression after cART initiation was defined as having two or more consecutive pVL tests <400 copies/mL spanning a period of at least six months.
